# Alterations of leptin in the course of inflammation and severe sepsis

**DOI:** 10.1186/1471-2334-12-217

**Published:** 2012-09-14

**Authors:** Michael Behnes, Martina Brueckmann, Siegfried Lang, Christian Putensen, Joachim Saur, Martin Borggrefe, Ursula Hoffmann

**Affiliations:** 1 First Department of Medicine, University Medical Centre Mannheim (UMM), Faculty of Medicine Mannheim, University of Heidelberg, Theodor-Kutzer-Ufer 1-3, 68167, Mannheim, Germany; 2 Department of Anesthesiology and Intensive Care Medicine, University of Bonn, Sigmund-Freud-Strasse 25, 53127, Bonn, Germany

**Keywords:** Adipocytes, Drotrecogin alpha (activated), Leptin, mRNA, Sepsis, Supernatants

## Abstract

**Background:**

The adipokine leptin regulates energy expenditure, vascular function, bone and cartilage growth as well as the immune system and systemic inflammatory response. Several activating effects towards T cells, monocytes, endothelium cells and cytokine production have been reported suggesting a protective role of leptin in the setting of an acute systemic inflammation. However, the pathophysiological role of leptin during severe sepsis is currently not elucidated in detail. This study aims to investigate leptin expression in cultured human adipocytes within an inflammatory model and in patients suffering from severe sepsis and evaluates treatment effects of drotrecogin alpha (activated) (DAA), the recombinant form of human activated protein C.

**Methods:**

In an *in-vitro* inflammatory model of adipocyte cell-culture the effect of DAA on leptin mRNA expression was evaluated. Synthesis of mRNA was measured by quantitative polymerase chain reaction (qPCR). Additionally, supernatants of these adipocytes as well as serum levels of adiponectin were measured in blood of 104 severe septic patients by ELISA-method. 26 patients were treated with DAA (DAA+), 78 patients were not treated with DAA (DAA-).

**Results:**

Stimulation of human adipocytes with TNF alpha over 6 and 24 hours resulted in a significant decrease by 46% and 59% of leptin mRNA transcripts compared to un-stimulated controls (p < 0.05). Leptin levels of supernatants of adipocyte culture decreased by 25% and 23% (p < 0.05) after incubation with TNF alpha after 6 and 24 hours. Incubation with DAA at 50 ng/ml DAA and 5 μg/ml doubled mRNA expression significantly at 24 hours (p < 0.05) but not at 6 hours. From day 1 to day 3 of sepsis, leptin levels increased in DAA+ compared to DAA- patients (p<0.10).

**Conclusions:**

Leptin appears to be involved in the pathogenesis of a systemic inflammatory response during sepsis. Administration of DAA significantly increased leptin expression. The specific mechanism or even benefit of DAA towards leptin needs further ongoing research.

## Background

Leptin belongs to the so-called adipokines, hormones being secreted in adipocytes of white adipose tissue [1]. Leptin interacts with specific ob-receptors and reveals pleiotropic neurohumoral functions, which regulate appetite and energy expenditure via the hypothalamic-pituitary-adrenal axis, vascular function, bone and cartilage growth, pregnancy as well as the regulation of the immune system and systemic inflammatory response [2]. Several activating effects towards T cells, monocytes, endothelium cells and cytokine production have been reported and might suggest both a pro-inflammatory and protective role for leptin in the setting of an acute systemic inflammation [3], [4].

Sepsis is defined as a clinical syndrome including an infection and the systemic inflammatory response [5], [6]. Because of its high incidence, mortality rate and associated costs within the healthcare system sepsis has become a major challenge of today´s medicine [7], [8]. Sepsis emerges to severe sepsis when one or more organ dysfunctions reemerge. Individual factors (eg, genetic factors or premorbid health status), extent of infection, and complicating organ dysfunction have a substantial impact on the disease process. Several inflammatory and procoagulant markers have been identified indicating the disease severity in these patients (eg, procalcitonin (PCT), interleukin 6 (IL-6), D-dimer) [5], [6], [9].

However, the role of leptin during sepsis is not yet fully elucidated. Conflicting results have been reported in which leptin was supposed to be either up- or down-regulated or even unchanged during systemic inflammation [10-13]. In addition to its predominant role in the inhibition of blood coagulation, human activated protein C (APC) plays an important role in the regulation of inflammation [14]. Multiple biological activities of APC have been demonstrated both in clinical and experimental settings, comprising pro-fibrinolytic, immune-modulating and anti-apoptotic properties [15-21]. Drotrecogin alpha (activated) (DAA) is the recombinant form of human activated protein C which was administered patients in our intensive care unit. Therefore we additionally analysed DAA in our inflammatory cell model for possible modulatory effects on leptin levels.

This study aims to investigate the expression of leptin both – *in-vitro* – in human adipocyte cell-culture within an inflammatory model and – *in vivo* – in patients suffering from severe sepsis, thereby additionally evaluating the specific treatment effect of drotrecogin alpha (activated) (DAA) on leptin expression.

## Methods

### Cell culture of human adipocytes

Cryopreserved Human White Preadipocytes (HWP) from subcutaneous adipose tissue and all culture media were supplied by Promocell (Heidelberg, Germany). They were cultivated as described by Promocell at 37°C in a humidified atmosphere of 95% air / 5% CO_2_. Medium was changed every 2–3 days. Preadipocytes were plated at a density of 5000 cells/cm [2] and cultured to total confluency stage in 6-well plates with 3 mL growth medium without antibiotics, containing 5% fetal calf serum. Growth medium was replaced and differentiation medium (including d-Biotin 8 μg/mL, Insulin 0.5 μg/mL, Dexamethasone 400 pg/ml, IBMX 44 μg/mL, L-Thyroxine 9 pg/ml, Ciglitazone 3 μg/mL) was added for 72 h to start the differentiation process. To complete differentiation the cells were then cultured for further 12 days with adipocyte nutrition medium (including fetal calf serum 3%, d-Biotin 8 μg/mL, Insulin 0.5 μg/mL, Dexamethasone 400 pg/ml). During this time lipid droplets developed. Fully differentiated cells at day 15 after starting the differentiation process were preincubated with 1 mL nutrition medium lacking fetal calf serum and Dexamethasone for 24 h before starting the experiment. The dosages of TNF alpha and DAA used for the present analyses accorded to our established protocols within our working group, which have formerly been published, respectively in an inflammatory model with endothelial cells [20], [21]. Several dosages of the highly selective and efficient stimulator TNF alpha resulted in 1 ng/ml as a concentration leading to sub-maximal effects on cytokines like IL-8 or IL-6. Drotrecogin alpha (activated) was added to yield final concentrations of 50 ng/ml and 5 μg/ml. 1 h later TNF alpha was accordingly added for 1 ng/ml. Cells were incubated for 6 h and 24 h and harvested to prepare total RNA. Supernatants of cell medium were frozen for quantification of leptin by ELISA. Treatment with DAA (50 ng/ml or 5 μg/ml) and TNF alpha (1 ng/ml) did not affect adipocyte viability, as assessed by trypan blue exclusion (90% viable cells, no difference to untreated controls).

### mRNA expression of leptin

For quantitative evaluation of DAA-dependent leptin mRNA steady-state expression in adipocyte cultures, total RNA was prepared by using the RNeasy mini column kit (Qiagen, Hilden, Germany), including DNAse treatment. Three micrograms of RNA were reverse transcribed and converted to cDNA with oligo(dT)_15_ primers using AMV reverse transcriptase according to standard protocols (Roche Applied Science, Germany, AMV Cat. No. 11495062001). The cDNA was amplified by quantitative PCR (qPCR) on the ABI 7000 realtime system (Applied Biosystems, Foster City, USA) using a qPCR-mix with hot start Taq DNA polymerase, SYBR-Green and enzyme system which reduces carryover DNA contamination (SYBR® GreenER™, Invitrogen, Cat.No.11760500) in the presence of sense and antisense primers (400 nM each). The sense- and antisense-primers for leptin were supplied from SABiosciences Corp. (Cat. No. PPH00581A). GAPDH as housekeeping gene was analyzed using primers as follows: [22] sense 5‘-TGCACCACCAACTGCTTAGC-3‘, antisense 5‘-GGCATGGACTGTGGTCATGAG-3‘. The qPCR condition consisted of 50°C for 2 min (reduction of DNA carryover contamination), 95°C for 10 min followed by 40 cycles of 95°C for 15 sec and 60°C for 1 min, followed by melting-curve analysis to verify the correctness of the amplicon. Relative quantification of leptin mRNA expression was calculated as follows: The expression of leptin mRNA relative to the housekeeping gene GAPDH in samples from cells treated with DAA or untreated (Control) was calculated by the ΔΔCT method, based on the threshold cycle (CT), as fold change = 2^−Δ(ΔCT)^, where ΔCT = CT_leptin_ − CT_GAPDH_ and Δ(ΔCT) = ΔCT_DAA_ − ΔCT _Control_[23], [24]. Efficiencies of the amplification reactions were calculated with a typical sample analysing the slope of the regression line of a 10-fold dilution series of cDNA (log10) versus CT: Efficiency =10^-1/slope^. Only primer pairs that showed an amplification efficiency between 1.90 – 2.05 (90 –105%) and a coefficient of correlation (r) between 0.90 – 1.0 were used for quantification [25]. For verification of the correct length of amplification products, they were analysed on an ethidium bromide stained 2% agarose gel. All cell-culture experiments have been repeated at least twice to guarantee repeatability.

### Study population

A total of 104 patients suffering from severe sepsis were prospectively enrolled at the First Department of Medicine, University Medical Centre Mannheim (UMM) (Germany) and at the Department of Anaesthesiology and Intensive Care Medicine, University Hospital Bonn (Germany) from March 2001 until October 2006. The study was carried out according to the principles of the declaration of Helsinki and was approved by the medical ethics commission II of the Medical Faculty Mannheim, University of Heidelberg, Germany. Written informed consent was obtained from all participating patients or their legal representatives.

The diagnosis of severe sepsis was based on criteria established by the American College of Chest Physicians and the Society of Critical Care Medicine Consensus Conference 1992: [5] Patients presented with a proven infection, three or more criteria of the systemic inflammatory response syndrome (SIRS criteria) and at least 1 of the following newly developed, sepsis-induced organ failures: cardiovascular organ failure with need for vasopressors, pulmonary organ failure defined as PaO_2_/FiO_2_ <250, renal organ failure with urine output <0,5 ml/kg/h, hematological organ failure with platelet count <80000/mm [3] or an unexplained metabolic acidosis with pH <7.3 and lactate levels >1.5 times of the upper limit of normal. Sepsis-induced organ failures in these patients were strongly connected to infection and were present for less than 24 h. Severity of sepsis was defined by the Acute Physiology and Chronic Health Evaluation II (APACHE II) score [26].

Out of a total of 104 patients suffering from severe sepsis 26 patients were treated with drotrecogin alpha (activated) (DAA) and 78 patients were not treated. The decision to treat patients with DAA or without was based on site-specific treatment modalities concerning the inclusion and exclusion criteria for the administration of DAA [27]. Twenty-six septic patients received a 96-hour infusion of DAA (XIGRIS®: 24 *μ*g per 1 kg body weight per hour). 78 patients with severe sepsis did not receive DAA because of contraindications for drotrecogin alpha (activated) such as platelet count 30000/mm, need for therapeutic anticoagulation with heparin, increased risk of bleeding, recent gastrointestinal bleeding, or stroke within the last 3 months [28]. Additionally 45 healthy humans were included to serve as a control group.

Baseline characteristics, such as APACHE II scores, body mass index (BMI), creatinine levels, white blood cell count, platelet count, C-reactive protein (CRP), bilirubin, international normalized ratio (INR), activated partial thromboplastin time (a-PTT), antithrombin III (AT III), D-dimer as well as body temperature were determined on day 1 of severe sepsis. Total clinical follow-up lasted over 28 days and defined clinical outcomes such as death or survival were documented.

### Plasma samples and leptin assay

Blood samples were obtained by venipuncture into serum monovettes® within the first 24 h after the diagnosis of severe sepsis (day 1), as well as on day 3 and 5 of severe sepsis. Within 30 minutes all blood samples were centrifuged at 1000 x *g* at 4°C for 15 minutes. Serum was separated, frozen and stored at −80°C.

Leptin measurement was performed with a solid phase two-site immunosorbent assay (ELISA) (Quantikine® human leptin immunoassay, R&D Systems Inc., Minneapolis, USA) [29].

### Statistical analysis

For data with normal distribution, the Student *t* test was applied. Otherwise, the Mann–Whitney *U* test was used as a nonparametric test. Deviations from a Gaussian distribution were tested by the Kolmogorov-Smirnov test. To generate hypotheses we used multiple t-tests with Holm-Bonferroni adjustments. For cell culture experiments the most important groups were specified in advance to be compared by statistical analysis: i) Control versus TNF alpha; ii) TNF alpha versus TNF alpha + DAA 50 ng/mL; iii) TNF alpha + DAA 50 ng/mL versus TNF alpha + DAA 5 μg/mL. Pearson’s correlation was used for normally distributed data. Spearman’s rank correlation was used for nonparametric data. Non-continuous variables were analyzed by the use of a 2x2 table and Fisher’s exact test. Data are presented as mean ± standard deviation (SD) or standard error of mean (SEM) as indicated. Values of P < 0.10 (two-tailed) were considered tending to be significant and P < 0.05 (two-tailed) were considered statistically significant. The calculations were performed with InStat (GraphPad Software) and SPSS software (SPSS Software GmbH).

## Results

### *In-vitro* results

**Figure 1:**
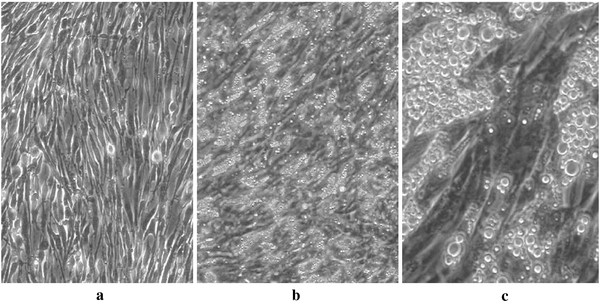
**Confluent cultures of undifferentiated preadipocytes (a) and mature adipocytes after differentiation for 15 days (b,c).** The high amount of fat droplets, visible as light globules (b,c), demonstrates the cells being differentiated to mature adipocytes. Cells were photographed with 100x **(a,b)** and 400x **(c)** magnification.

**Figure 2:**
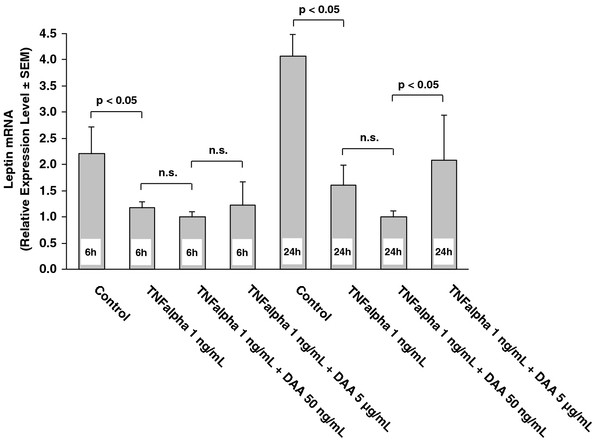
**Leptin mRNA expression was significantly decreased after 6 and 24 hours of stimulation with 1 ng/ml TNF alpha (p < 0.05) in human adipocytes.** Drotrecogin alpha (activated) (DAA) dose-dependently increased leptin mRNA levels after 24 hours from DAA 50 ng/ml to DAA 5 μg/ml (p < 0.05), but at 6 hours no significant change could be detected. Significances (n.s. = not significant) of t-tests with Holm-Bonferroni adjustments for multiple comparisons are indicated. Only the relevant in advance specified group comparisons were analysed. Data are presented as mean with standard error of mean (SEM).

*In-vitro* analyses showed final cell differentiation of human adipocytes after 15 days of cell growth (Figure 1a-c). Stimulating human adipocytes with tumor necrosis factor alpha alone (concentration TNF alpha, 1 ng/ml) over 6 hours resulted in leptin mRNA transcripts decreased by 46% compared to un-stimulated controls (p < 0.05) (Figure 2). Adding DAA at 50 ng/ml or 5 μg/ml to TNF alpha at 1 ng/ml, a significant change of leptin mRNA expression could not be detected (Figure 2). Stimulating human adipocytes with TNF alpha alone at the same concentration of 1 ng/ml for a longer time of 24 hours resulted in leptin mRNA transcripts decreased by 59% compared to un-stimulated controls (p < 0.05) (Figure 2). Adding DAA at 50 ng/ml or 5 μg/ml to TNF alpha at 1 ng/ml, a significant upregulation of leptin mRNA expression was detected after 24 hours from DAA at 50 ng/ml to 5 μg/ml (p < 0.05) (Figure 2).

**Figure 3:**
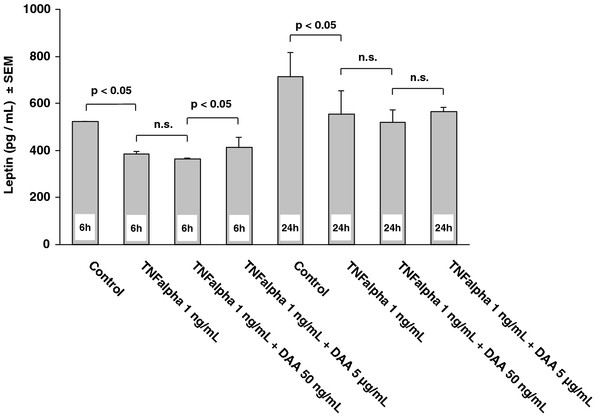
**Leptin levels out of supernatants of the adipocytes cell culture measured by ELISA showed decreased levels of leptin after incubation with TNF alpha for 6 and 24 hours (p < 0.05).** Increasing levels of leptin were observed after 6 hours of incubation according to the applied dosages of DAA (50 ng/ml and 5 μg/ml). Significances (n.s. = not significant) of t-tests with Holm-Bonferroni adjustments for multiple comparisons are indicated. Only the relevant in advance specified group comparisons were analysed. Data are presented as mean with standard error of mean (SEM).

Similar expression profiles as with leptin mRNA were seen when measuring leptin levels out of supernatants of the adipocytes by ELISA. Leptin levels of supernatants of adipocyte culture decreased by 25% (p < 0.05) after incubation with TNF alpha alone after 6 hours (Figure 3). After 6 hours incubation with TNF alpha at 1 ng/ml and DAA at 50 ng/ml or 5 μg/ml leptin levels increased from DAA at 50 ng/ml to 5 μg/ml (p < 0.05) (Figure 3). After 24 hours leptin levels significantly decreased by 23% after incubation with TNF alpha alone (p < 0.05) (Figure 3). But a significant change of leptin levels from DAA at 50 ng/ml to 5 μg/ml could not be detected after 24 hours (Figure 3).

Treatment with DAA (50 ng/ml or 5 μg/ml) and TNF alpha (1 ng/ml) did not affect adipocyte viability, as assessed by trypan blue exclusion (90% viable cells, no difference to untreated controls; data not shown).

### *In-vivo* results

Baseline characteristics of 104 patients suffering from severe sepsis are given in Table 1. Mean age of all study patients was 64 years. Patients treated with DAA (n = 26) were younger than patients not treated (n = 78) (57 years vs. 67 years; p = 0.002). The most common primary site of infection was the lung (n = 51), followed by cardiac (n = 12) and intra-abdominal infections (n = 7) (p = 0.3). The mean APACHE II score was 28.3 (SD = 5.4). Mean BMI levels were similar in all patient groups (DAA+, mean = 25.2, ±SEM = 2.5, DAA-, mean = 26.6, ±SEM = 0.9, p = 0.6).

**Table 1:** Baseline characteristics of 104 patients suffering from severe sepsis at day 1

	**All patients (n = 104)**	**DAA + (n = 26)**	**DAA − (n = 78)**	**p value †**
**Age** (years)*	64.0 ± 13.5	56.6 ± 15.0	66.5 ± 12.1	0.002
**Gender** (n)				
Male	62	16	46	0.8
Female	42	10	32	
**Body mass index (BMI)** *	26.4 ± 0.8	25.2 ± 2.5	26.6 ± 0.9	0.6
**Heart rate** (beats/min) *	84 ± 23.6	85 ± 20.8	84 ± 24.5	0.76
**APACHE II score** *	28.3 ± 5.4	28.6 ± 6.5	28.1 ± 5.0	0.9
**Primary site of infection** (n)				
Lung	51	10	41	
Urinary	6	2	4	
Cerebral	5	2	3	
Cardiac	12	4	8	0.3
Intra-abdominal	7	-	7	
Skin	2	-	2	
Unknown	21	8	13	
**CRP** (mg/l)*	164 ± 126	206 ± 151	151 ± 115	0.2
**Creatinine** (mg/dl)*	2.6 ± 2.1	3.0 ± 2.3	2.5 ± 2.0	0.2
**White blood cells** (10^9^/l)*	16.4 ± 8.0	16.0 ± 9.7	16.5 ± 7.5	0.4
**Platelets** (10^9^/l)*	210 ± 117	202 ± 115	213 ± 118	0.5
**aPTT** (sec)*	40.7 ± 24.4	42.2 ± 25.5	40.2 ± 24.2	0.4
**Antithrombin III** (%)*	63.5 ± 19.5	61.4 ± 17.1	64.3 ± 20.6	0.8
**D-dimer** (μg/ml)*	5.0 ± 3.8	3.0 ± 2.0	5.5 ± 4.1	0.4
**Bilirubin** (mg/dl)*	1.7 ± 1.8	2.6 ± 2.3	1.4 ± 1.5	0.04
**Death** (n)	53	17	36	0.9

* Data are presented as mean ± standard deviation (SD).† p values are calculated for the comparison between DAA+ patients with DAA- patients.

Leptin serum levels significantly correlated with age, body temperature, BMI and APACHE II score. Additionally leptin levels inversely correlated with adiponectin, and correlated positively with white blood cells, antithrombin III, interleukin 6 and creatinine (Table 2). However, there were no significant differences of leptin levels in patients with impaired renal function (defined as a serum creatinine value above 1.1 mg/dl) compared to patients with a regular renal function (defined as a serum creatinine value below or equal than 1.1 mg/dl) (patients with renal failure, leptin mean 29282 pg/ml, ± SEM 4398 pg/ml, n = 75; patients without renal failure, leptin mean 21896 pg/ml, ± 5631 pg/ml, n = 29; p = 0.3). No association was found between leptin and C reactive protein (Table 2).

**Table 2:** Correlations between leptin levels and clinical indices at day 1 of severe sepsis

	**All patients (n = 104)**		**DAA(n = 26)**		**No DAA(n = 78)**	
	**r**	**p-value**	**r**	**p-value**	**r**	**p-value**
Age	0.25	**0.01**	0.34	**0.09**	0.26	**0.02**
Body temperature	0.27	**0.007**	0.57	**0.005**	0.28	**0.02**
Body mass index (BMI)	0.40	**0.01**	0.49	0.4	0.41	**0.01**
APACHE II score	0.28	**0.03**	0.34	0.15	0.32	**0.04**
Adiponectin	−0.28	**0.04**	−0.26	0.19	−0.33	**0.003**
White blood cell count	0.19	**0.06**	−0.14	0.52	0.20	**0.08**
CRP	−0.06	0.57	0.15	0.49	−0.09	0.46
Interleukin 6	−0.09	0.37	0.53	**0.009**	−0.02	0.88
Hemoglobin	0.19	**0.06**	0.44	**0.03**	0.12	0.28
Hematocrit	0.20	**0.04**	0.46	0.02	0.15	0.20
Platelets	0.21	**0.03**	0.16	0.4	0.15	0.18
Creatinine	0.22	**0.03**	0.12	**0.6**	0.32	**0.005**
Antithrombin III	0.31	**0.03**	0.6	**0.02**	0.35	**0.04**

**Figure 4:**
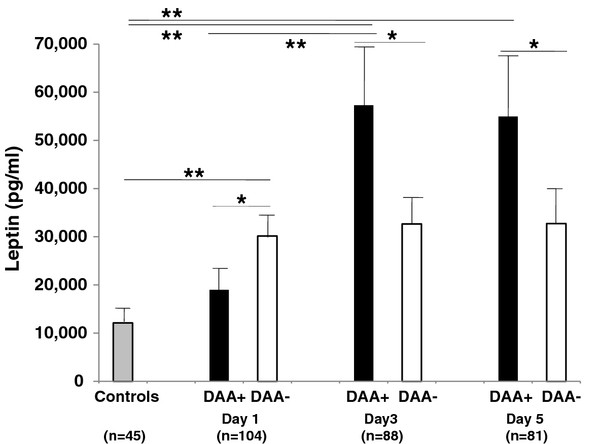
**Treating patients with DAA resulted in an increase in leptin levels after 96 hours of DAA infusion compared to patients without treatment of DAA from day 1 to day 3 and 5 (p < 0.05) (leptin concentrations, Day 1: DAA+, mean = 18991 pg/ml, ±SEM = 6009 pg/ml. Day 3: DAA+, mean = 57315 pg/ml, ± SEM = 17075 pg/ml. Day 5: DAA+, mean = 54980 pg/ml, ±SEM = 17660 pg/ml).** Significances of t-tests with Holm-Bonferroni adjustments for multiple comparisons are indicated, otherwise no significance could be reached. Data are presented as mean with standard error of mean (SEM). * p < 0.10, ** p < 0.05.

As demonstrated in Figure 4, patients suffering from severe sepsis without treatment of DAA revealed significantly higher serum concentrations of leptin compared to healthy controls (n = 45, mean = 12116 pg/ml, ± SEM = 1945 pg/ml) (leptin concentrations, Day 1: DAA-, mean = 30175 pg/ml, ±SEM = 4203 pg/ml. Day 3: DAA-, mean = 32670 pg/ml, ± SEM = 6173 pg/ml. Day 5: DAA-, mean = 32731 pg/ml, ±SEM = 6220 pg/ml) (p < 0.05).

At the beginning of treatment with DAA (day 1) leptin levels showed a tendency (p < 0.10) to be lower (day 1: DAA+, mean = 18991 pg/ml, ±SEM = 6009 pg/ml, nc = 26) compared to patients without DAA (Figure 4). From day 1 to day 3 of sepsis, leptin levels increased in patients treated with DAA compared to patients without treatment of DAA (p < 0.10) (leptin concentrations, Day 1: DAA+, mean = 18991 pg/ml, ±SEM = 6009 pg/ml. Day 3: DAA+, mean = 57315 pg/ml, ± SEM = 17075 pg/ml. Day 5: DAA+, mean = 54980 pg/ml, ±SEM = 17660 pg/ml).

There was no difference of leptin concentrations between patients surviving 28 days follow-up (mean = 26073 pg/ml, ±SEM = 4704 pg/ml, n = 51) compared to those who were deceased (mean = 28824 pg/ml, ±SEM = 5442 pg/ml, n = 53) (p > 0.10).

## Discussion

This study investigated the expression of leptin both – *in vitro* – within an inflammatory model of human cultured adipocytes and – *in vivo* – in patients suffering from severe sepsis and additionally evaluated the specific treatment effect of drotrecogin alpha (activated) (DAA).

Leptin mRNA and protein expression was down-regulated by TNF alpha after 6 and 24 hours of incubation in human adipocytes. But this effect seems not to be due to a reduced number of viable cells because of TNF alpha treatment: Treatment with DAA (50 ng/ml or 5 μg/ml) and TNF alpha (1 ng/ml) did not affect adipocyte viability, as assessed by trypan blue exclusion. Moreover, administration of DAA (50 ng/ml or 5 μg/ml) in the presence of TNF alpha (1 ng/ml) up-regulated leptin mRNA and protein expression in a dose dependent way at 24 hours. In patients suffering from severe sepsis leptin serum levels remained at the same level from days 1 to 5 in those patients without treatment of DAA. However, in patients treated with DAA leptin serum levels increased from day 1 to day 3 day of severe sepsis during the infusion of DAA over 96 hours. Leptin serum levels were inversely correlated with adiponectin and positively correlated with APACHE II score, body temperature, white blood cell count and interleukin 6.

The present analysis confirmed preliminary reports demonstrating decreased mRNA expression of leptin following TNF alpha stimulation within an *in-vitro* inflammatory model [30-34]. However, conflicting results have been obtained from cell cultures demonstrating an up-regulation of leptin mRNA expression after stimulation with TNF alpha [35], [36]. These varieties might in part be explained by the use of different cell species, anatomical localization of the fat tissue and the duration of exposure to the stimulating cytokines [32]. Hence, mRNA transcription was even more down-regulated after longer incubation of 24 hours compared to 6 hours (59% vs. 46%).

In accordance with recently published data we could demonstrate increasing levels of leptin in patients suffering from severe sepsis compared to healthy controls [11], [37-41] and showed several correlations with established clinical markers of sepsis. Therefore our observations support evidence that leptin appears to be involved in the pathogenesis and the course of a systemic inflammatory response during sepsis. Interestingly, with regard to the body of literature it was shown that leptin itself increases the production of TNF alpha or Interleukin 6 from macrophages [42].

DAA has been shown to reveal antithrombotic, profibrinolytic, anti-inflammatory and antiapoptotic properties [27], [28]. However, no data currently exist, whether DAA influences the release of the adipokine leptin. To the best of our knowledge this study is the first to investigate the influence of DAA on leptin expression. It could have been demonstrated that administration of DAA increases the amount of leptin mRNA and protein expression over time in human adipocytes and furthermore significantly increases leptin serum levels in patients suffering from severe sepsis after 96 hours administration of DAA.

Therefore, the present findings might reveal another effect of DAA during sepsis, i.e. the up-regulation of the hormone leptin under circumstances of ongoing systemic inflammation. Several in-vitro studies already demonstrated specific anti-inflammatory effects of DAA, e.g. inhibition of transcription factor NF-kappa B in isolated mononuclear cells or the inhibition of macrophage inflammatory protein-1 (MIP-1-alpha), monocyte chemoattractant protein-1 (MCP-1), neopterin [19], [43], [44] or up-regulation of adiponectin [45]. However, the role of specifically leptin during severe sepsis is not yet completely understood. It could have been demonstrated that patients surviving an acute sepsis revealed increased levels of leptin, whereas leptin correlated with the disease severity and is an independent predictor of death [37], [38], [41]. Shapiro et al. [46] postulated that leptin might be able to pronounce endothelial dysfunction during sepsis and therefore worsens outcome of the disease. However, increases of the soluble leptin receptor (sLR) were explained as a compensatory anti-inflammatory mechanism.

### Limitations

The exact mechanisms contributing to increased leptin mRNA and protein expressions after administration of DAA in human adipocytes and patients suffering from severe sepsis were not determined in this study. However, we performed cell culture experiments with adipocytes in order to confirm our clinical findings in septic patients with a more straightforward system and proving the involvement of adipocytes. Instead of LPS we used TNF alpha as a more selective and efficient pro-inflammatory stimulus for sepsis according to our established protocols, respectively in an inflammatory model with endothelial cells having been published already [20], [21]. It was our intention to apply the same conditions as in the above mentioned inflammatory models in order to guarantee comparability of our own experimental approach. However, TNF alpha serum levels were not measured in our septic patients. Regarding clinical benefits of septic patients increased leptin levels could not show a survival benefit over 28-days follow-up in our study. Nevertheless, our results need to be confirmed by ongoing basic research analyses and larger prospective clinical studies to explain the more detailed mechanisms beyond our findings.

## Conclusions

Taken together, it has been demonstrated that leptin mRNA and -protein expression is down-regulated by TNF alpha in human adipocytes. Administration of DAA up-regulated leptin expression over time and increasing applied dosages of DAA. Accordingly, patients suffering from severe sepsis revealed increased leptin serum levels, while patients treated with DAA revealed increasing leptin serum levels after complete treatment with DAA (i.e. 96 hours infusion) at day 3 and 5 compared to untreated septic patients. Therefore, our results support the hypothesis that leptin might be involved in the pathogenesis of a systemic inflammatory response during sepsis possibly acting as an anti-inflammatory protein. The specific mechanism or even benefit of DAA administration in the time course of sepsis towards leptin needs further ongoing research.

## Abbreviations

APACHE II: Acute Physiology and Chronic Health Evaluation II; aPTT: Activated partial thromboplastin time; AT III: Antithrombin III; BMI: Body mass index; CRP: C-reactive protein (CRP); CVD: Central venous pressure; DAA: Drotrecogin alpha (activated); ELISA: Enzyme-Linked Immunosorbent Assay; ICU: Intensive care unit; INR: International normalized ratio; LPS: Lipopolysaccheride; mRNA: Messenger ribonucleic acid; qPCR: Quantitative polymerase chain reaction; SD: Standard deviation; SEM: Standard error of mean; TNF alpha: Tumor necrosis factor alpha.

## Competing interests

The authors declare that they have no competing interests. This study is part of a registered clinical trial (ClinicalTrials.gov Identifier: NCT00222222).

## Author’s information

†Martina Brueckmann is an employee of Boehringer Ingelheim GmbH & Co.KG and a lecturer of the Medical Faculty Mannheim, University of Heidelberg, Germany.

## Authors’ contributions

MBe: study design, data analysis, manuscript preparation and review; SL: carried out the cell culture experiments, data analysis and manuscript review; MBr, JS, MBo: study advice, manuscript review; CP: study design and manuscript review; UH: conceived the study, enrolled study patients and reviewed the manuscript. All authors read and approved the final manuscript.

## Pre-publication history

The pre-publication history for this paper can be accessed here:

http://www.biomedcentral.com/1471-2334/12/217/prepub

## References

[B1] Trayhurn P Wood IS Adipokines: Inflammation and the pleiotropic role of white adipose tissue Br J Nutr 2004 92 347 355 10.1079/BJN20041213 15469638

[B2] Lago F Dieguez C Gomez-Reino J Gualillo O The emerging role of adipokines as mediators of inflammation and immune responses Cytokine Growth Factor Rev 2007 18 313 325 10.1016/j.cytogfr.2007.04.007 17507280

[B3] Fantuzzi G Adipose tissue, adipokines, and inflammation J Allergy Clin Immunol 2005 115 911 919 quiz 920 10.1016/j.jaci.2005.02.023 15867843

[B4] Otero M Lago R Gomez R Dieguez C Lago F Gomez-Reino J Gualillo O Towards a pro-inflammatory and immunomodulatory emerging role of leptin Rheumatology (Oxford) 2006 45 944 950 10.1093/rheumatology/kel157 16720637

[B5] Bone RC Balk RA Cerra FB Dellinger RP Fein AM Knaus WA Schein RM Sibbald WJ Definitions for sepsis and organ failure and guidelines for the use of innovative therapies in sepsis. The accp/sccm consensus conference committee. American college of chest physicians/society of critical care medicine Chest 1992 101 1644 1655 10.1378/chest.101.6.1644 1303622

[B6] Levy MM Fink MP Marshall JC Abraham E Angus D Cook D Cohen J Opal SM Vincent JL Ramsay G 2001 sccm/esicm/accp/ats/sis international sepsis definitions conference Crit Care Med 2003 31 1250 1256 10.1097/01.CCM.0000050454.01978.3B 12682500

[B7] Angus DC Linde-Zwirble WT Lidicker J Clermont G Carcillo J Pinsky MR Epidemiology of severe sepsis in the united states: Analysis of incidence, outcome, and associated costs of care Crit Care Med 2001 29 1303 1310 10.1097/00003246-200107000-00002 11445675

[B8] Angus DC Pereira CA Silva E Epidemiology of severe sepsis around the world Endocr Metab Immune Disord Drug Targets 2006 6 207 212 16787296 10.2174/187153006777442332

[B9] Dhainaut JF Shorr AF Macias WL Kollef MJ Levi M Reinhart K Nelson DR Dynamic evolution of coagulopathy in the first day of severe sepsis: Relationship with mortality and organ failure Crit Care Med 2005 33 341 348 10.1097/01.CCM.0000153520.31562.48 15699837

[B10] Koch A Weiskirchen R Zimmermann HW Sanson E Trautwein C Tacke F Relevance of serum leptin and leptin-receptor concentrations in critically ill patients Mediators Inflamm 2010 pii:473540 10.1155/2010/473540 PMC2943118 20871818

[B11] Yousef AA Amr YM Suliman GA The diagnostic value of serum leptin monitoring and its correlation with tumor necrosis factor-alpha in critically ill patients: A prospective observational study Crit Care 2010 14 R33 10.1186/cc8911 20230641 PMC2887140

[B12] Hillenbrand A Knippschild U Weiss M Schrezenmeier H Henne-Bruns D Huber-Lang M Wolf AM Sepsis induced changes of adipokines and cytokines - septic patients compared to morbidly obese patients BMC Surg 2010 10 26 10.1186/1471-2482-10-26 20825686 PMC2944119

[B13] Langouche L Vander Perre S Frystyk J Flyvbjerg A Hansen TK Van den Berghe G Adiponectin, retinol-binding protein 4, and leptin in protracted critical illness of pulmonary origin Crit Care 2009 13 R112 10.1186/cc7956 19589139 PMC2750156

[B14] Esmon CT Taylor FB Snow TR Inflammation and coagulation: Linked processes potentially regulated through a common pathway mediated by protein c Thromb Haemost 1991 66 160 165 1833850

[B15] Joyce DE Grinnell BW Recombinant human activated protein c attenuates the inflammatory response in endothelium and monocytes by modulating nuclear factor-kappab Crit Care Med 2002 30 S288 293 10.1097/00003246-200205001-00019 12004250

[B16] Cheng T Liu D Griffin JH Fernandez JA Castellino F Rosen ED Fukudome K Zlokovic BV Activated protein c blocks p53-mediated apoptosis in ischemic human brain endothelium and is neuroprotective Nat Med 2003 9 338 342 10.1038/nm826 12563316

[B17] Brueckmann M Huhle G Max M [mechanisms of action of recombinant human activated protein c] Anaesthesist 2006 55 Suppl 1 5 15 16520928 10.1007/s00101-006-1001-z

[B18] Brueckmann M Hoffmann U De Rossi L Weiler HM Liebe V Lang S Kaden JJ Borggrefe M Haase KK Huhle G Activated protein c inhibits the release of macrophage inflammatory protein-1-alpha from thp-1 cells and from human monocytes Cytokine 2004 26 106 113 10.1016/j.cyto.2004.01.004 15135804

[B19] Brueckmann M Hoffmann U Dvortsak E Lang S Kaden JJ Borggrefe M Haase KK Drotrecogin alfa (activated) inhibits nf-kappa b activation and mip-1-alpha release from isolated mononuclear cells of patients with severe sepsis Inflamm Res 2004 53 528 533 10.1007/s00011-004-1291-z 15597147

[B20] Brueckmann M Horn S Lang S Fukudome K Schulze Nahrup A Hoffmann U Kaden JJ Borggrefe M Haase KK Huhle G Recombinant human activated protein c upregulates cyclooxygenase-2 expression in endothelial cells via binding to endothelial cell protein c receptor and activation of protease-activated receptor-1 Thromb Haemost 2005 93 743 750 15841323 10.1160/TH04-08-0511

[B21] Brueckmann M Marx A Weiler HM Liebe V Lang S Kaden JJ Zieger W Borggrefe M Huhle G Konstantin Haase K Stabilization of monocyte chemoattractant protein-1-mrna by activated protein c Thromb Haemost 2003 89 149 160 12540965

[B22] Vandesompele J De Preter K Pattyn F Poppe B Van Roy N De Paepe A Speleman F Accurate normalization of real-time quantitative rt-pcr data by geometric averaging of multiple internal control genes Genome Biol 2002 3 RESEARCH0034 12184808 10.1186/gb-2002-3-7-research0034 PMC126239

[B23] Schmittgen TD Livak KJ Analyzing real-time pcr data by the comparative c(t) method Nat Protoc 2008 3 1101 1108 10.1038/nprot.2008.73 18546601

[B24] Livak KJ Comparative Ct method. ABI Prism 7700 Sequence Detection System. User Bulletin no. 2 PE Applied Biosystems 1997

[B25] Dieffenbach CW, Dveksler GS PCR primer, a laboratory manual 2003 2 Cold Spring Harbor Laboratory Press, New York

[B26] Knaus WA Draper EA Wagner DP Zimmerman JE Apache ii: A severity of disease classification system Crit Care Med 1985 13 818 829 10.1097/00003246-198510000-00009 3928249

[B27] Hughes M Recombinant human activated protein c Int J Antimicrob Agents 2006 28 90 94 10.1016/j.ijantimicag.2006.05.021 16837170

[B28] Bernard GR Vincent JL Laterre PF LaRosa SP Dhainaut JF Lopez-Rodriguez A Steingrub JS Garber GE Helterbrand JD Ely EW Fisher CJ Efficacy and safety of recombinant human activated protein c for severe sepsis N Engl J Med 2001 344 699 709 10.1056/NEJM200103083441001 11236773

[B29] Quantikine® human leptin Immunoassay 2009 R&D Systems Inc, Minneapolis, USA internet-address: http://www.rndsystems.com/pdf/dlp00.pdf

[B30] Fawcett RL Waechter AS Williams LB Zhang P Louie R Jones R Inman M Huse J Considine RV Tumor necrosis factor-alpha inhibits leptin production in subcutaneous and omental adipocytes from morbidly obese humans J Clin Endocrinol Metab 2000 85 530 535 10.1210/jc.85.2.530 10690850

[B31] Granowitz EV Transforming growth factor-beta enhances and pro-inflammatory cytokines inhibit ob gene expression in 3 t3-l1 adipocytes Biochem Biophys Res Commun 1997 240 382 385 10.1006/bbrc.1997.7663 9388486

[B32] Laharrague P Truel N Fontanilles AM Corberand JX Penicaud L Casteilla L Regulation by cytokines of leptin expression in human bone marrow adipocytes Horm Metab Res 2000 32 381 385 10.1055/s-2007-978658 11069201

[B33] Yamaguchi M Murakami T Tomimatsu T Nishio Y Mitsuda N Kanzaki T Kurachi H Shima K Aono T Murata Y Autocrine inhibition of leptin production by tumor necrosis factor-alpha (tnf-alpha) through tnf-alpha type-i receptor in vitro Biochem Biophys Res Commun 1998 244 30 34 10.1006/bbrc.1998.8199 9514868

[B34] Gottschling-Zeller H Birgel M Scriba D Blum WF Hauner H Depot-specific release of leptin from subcutaneous and omental adipocytes in suspension culture: Effect of tumor necrosis factor-alpha and transforming growth factor-beta1 Eur J Endocrinol 1999 141 436 442 10.1530/eje.0.1410436 10526261

[B35] Gerhardt CC Romero IA Cancello R Camoin L Strosberg AD Chemokines control fat accumulation and leptin secretion by cultured human adipocytes Mol Cell Endocrinol 2001 175 81 92 10.1016/S0303-7207(01)00394-X 11325518

[B36] Sarraf P Frederich RC Turner EM Ma G Jaskowiak NT Rivet DJ Flier JS Lowell BB Fraker DL Alexander HR Multiple cytokines and acute inflammation raise mouse leptin levels: Potential role in inflammatory anorexia J Exp Med 1997 185 171 175 10.1084/jem.185.1.171 8996253 PMC2196098

[B37] Arnalich F Lopez J Codoceo R Jim nez M Madero R Montiel C Relationship of plasma leptin to plasma cytokines and human survivalin sepsis and septic shock J Infect Dis 1999 180 908 911 10.1086/314963 10438392

[B38] Bornstein SR Licinio J Tauchnitz R Engelmann L Negrao AB Gold P Chrousos GP Plasma leptin levels are increased in survivors of acute sepsis: Associated loss of diurnal rhythm, in cortisol and leptin secretion J Clin Endocrinol Metab 1998 83 280 283 10.1210/jc.83.1.280 9435456

[B39] Maruna P Gurlich R Frasko R Haluzik M Serum leptin levels in septic men correlate well with c-reactive protein (crp) and tnf-alpha but not with bmi Physiol Res 2001 50 589 594 11829320

[B40] Nylen ES Alarifi AA Humoral markers of severity and prognosis of critical illness Best Pract Res Clin Endocrinol Metab 2001 15 553 573 10.1053/beem.2001.0169 11800523

[B41] Torpy DJ Bornstein SR Chrousos GP Leptin and interleukin-6 in sepsis Horm Metab Res 1998 30 726 729 10.1055/s-2007-978967 9930630

[B42] Loffreda S Yang SQ Lin HZ Karp CL Brengman ML Wang DJ Klein AS Bulkley GB Bao C Noble PW Lane MD Diehl AM Leptin regulates proinflammatory immune responses FASEB J 1998 12 57 65 9438411

[B43] Behnes M Brueckmann M Liebe V Liebetrau C Lang S Putensen C Borggrefe M Hoffmann U Levels of oxidized low-density lipoproteins are increased in patients with severe sepsis J Crit Care 2008 23 537 541 10.1016/j.jcrc.2008.09.002 19056019

[B44] Behnes M Brueckmann M Wiessner M Kettenmann E Liebetrau C Lang S Putensen C Borggrefe M Hoffmann U Time-course of neopterin levels in patients suffering from severe sepsis treated with and without drotrecogin-alpha (activated) Scand J Infect Dis 2008 40 503 508 10.1080/00365540701808978 18584538

[B45] Behnes M Brueckmann M Lang S Putensen C Saur J Borggrefe M Hoffmann U Alterations of adiponectin in the course of inflammation and severe sepsis Shock 2012 38 243 248 10.1097/SHK.0b013e318261e0dc 22744305

[B46] Shapiro NI Khankin EV Van Meurs M Shih SC Lu S Yano M Castro PR Maratos-Flier E Parikh SM Karumanchi SA Yano K Leptin exacerbates sepsis-mediated morbidity and mortality J Immunol 185 517 524 20519646 10.4049/jimmunol.0903975 PMC3997057

